# Detection of Different Classes of Fluorinated Anions at Ionic-Liquid Surfaces by Reactive-Atom Scattering Using Laser-Ablated Al Projectiles

**DOI:** 10.1021/acs.jpcc.5c08388

**Published:** 2026-02-12

**Authors:** Paul D. Lane, Naomi S. Elstone, Duncan W. Bruce, John M. Slattery, Matthew L. Costen, Kenneth G. McKendrick

**Affiliations:** † Institute of Chemical Sciences, School of Engineering and Physical Sciences, 3120 Heriot-Watt University, Edinburgh EH14 4AS, U.K.; ‡ Department of Chemistry, 8748 University of York, Heslington, York YO10 5DD, U.K.

## Abstract

Reactive-atom scattering (RAS) using laser-ablated aluminum projectiles has been applied to probe the exposure of fluorinated anions at ionic-liquid (IL) surfaces. Gas-phase AlF was detected by laser-induced fluorescence (LIF) following interaction of the Al plume with ILs containing bis­(trifluoromethylsulfonyl)­imide ([Tf_2_N]^−^), trifluoromethanesulfonate ([OTf]^−^), and tetrafluoroborate ([BF_4_]^−^) anions, paired with 1-ethyl-3-methylimidazolium ([C_2_mim]^+^) or 1-octyl-3-methylimidazolium ([C_8_mim]^+^) cations. Clear AlF signals were observed for all three fluorinated anions, though yields varied markedly, with relative intensities following the sequence [BF_4_]^−^ > [Tf_2_N]^−^ ≫ [OTf]^−^. Molecular dynamics (MD) simulations employing solvent-accessible surface area and a ball-drop algorithm provided quantitative predictions of F-atom outer-surface exposure, defined as the combined surface area of atoms directly accessible to a probe particle of specified radius, which were compared with experimental AlF yields. The reduction in F-atom exposure, qualitatively expected with an increase in cation alkyl-chain length, was predicted by MD for all three anions and observed in the AlF yields from salts with [BF_4_]^−^ and [Tf_2_N]^−^. However, even for these salts, there were a number of quantitative differences between the predictions of outer-surface exposure and AlF yields, which may partially be explained by penetration of the incident projectiles below the alkyl-chain layer present at the extreme outer surface of the liquids. Discrepancies for [OTf]^−^ salts were much larger and are most likely evidence for anion-specific competing primary reactions that suppress AlF production, or for secondary processes that prevent it from surviving and escaping into the gas phase. These results provide new insight into the subtlety of the reactions of the species in the Al plume with fluorinated anions and point to the further understanding that is needed to establish Al-ablation RAS-LIF as a quantitative probe of fluorinated species at IL interfaces.

## Graphical abstract

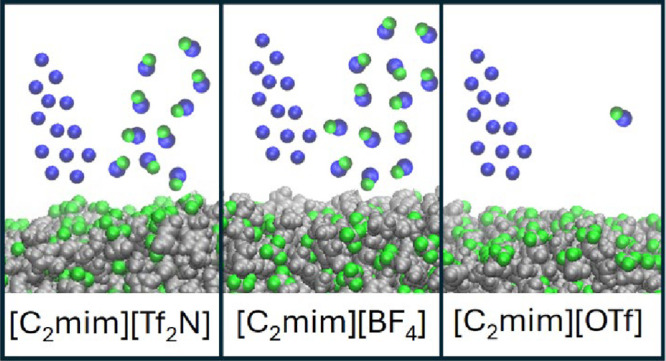

## Introduction

The gas–liquid interface plays a crucial role in numerous environmental and industrial processes. One category of liquids whose surfaces have been of particular interest in recent years is ionic liquids (ILs). ILs are often defined as salts that remain liquid below 100 °C. Their unique properties, such as low vapor pressure, thermal stability, solvation versatility, and broad electrochemical windows, have made them attractive for a wide range of applications.^1–7^ Among their many uses, surface properties of ILs are particularly critical in processes such as gas separation and multiphase catalysis where gas-phase molecules are accommodated at and transported through the gas–liquid interface.^2,5^ There are ongoing efforts to identify ILs best-suited for specific tasks such as these, and in parallel to devise surface-analytical methods to establish a rational basis for why certain materials provide improved performance.^8–10^ The expectation that the reaction medium will have a significant influence on catalytic performance derives in part from the well-known differential solubilities and transport properties of substrates and products in prototypical reactions such as hydrogenation or hydroformylation of small alkenes.^11–13^ It is also postulated that the surface activity of metal–ligand complexes in different media will affect their performance as homogeneous catalysts.^14–18^

ILs have been studied using a variety of classical and advanced techniques to better understand their interfacial composition and structure. Classical methods such as surface tension provide indirect insights, because the observed bulk quantity can only be related to surface composition through some form of modeling or fitting.^19^ More sophisticated approaches include neutron and X-ray reflectometry;^20–23^ although it is still the case that chemical composition must be inferred indirectly from a model of a secondary property (electron density or scattering-length density). Methods that are more intrinsically chemically specific include angle-resolved photoelectron spectroscopy (ARXPS);^24–27^ Rutherford backscattering (RBS);^28–30^ low-energy ion scattering (LEIS);^31–33^ neutral-impact-collision ion-scattering spectroscopy (NICISS);^34–37^ secondary-ion mass spectrometry (SIMS);^30,38^ metastable-atom electron spectroscopy (MAES)/metastable-induced electron spectroscopy (MIES);^37,39,40^ and nonlinear optical spectroscopies, particularly sum-frequency generation (SFG).^9,41–44^

The method that we apply here is a variant of reactive-atom scattering (RAS), which we have developed jointly with Minton and co-workers.^10,45–56^ In the original implementation of RAS and in most of its applications to-date in the characterization of IL surfaces, O atoms were used as the probe projectile. The scattered chemical products resulting from reaction at specific sites on the IL surface were detected either by mass spectrometry (in RAS-MS) or by laser-induced fluorescence (in RAS-LIF). In the ILs studied, the OH (and H_2_O detectable by RAS-MS) ‘reporter’ products can effectively only be produced in reaction with the alkyl chains present in the cationic component, thus providing chemical specificity. This has allowed the factors determining alkyl-chain exposure to have been established successfully in a wide range of IL materials, including the effects of alkyl chain length,^45–50^ the nature of the cation headgroup,^52^ the identity of the counterion,^49^ and, importantly, competition for surface sites in mixtures of ILs.^53,54,56^ Where available, these results are generally complemented by and corroborate those of other methods, at least at a qualitative level.

The surface sensitivity of RAS derives from similar principles to those of the other methods noted above that are based on exposing the liquid to an incident projectile (in a general sense, such as the reactive atom in RAS, or e.g. an ionizing photon (ARXPS) or incident ion (RBS, LEIS, NICISS, SIMS) or metastable atom (MAES/MIES)). In general, it results from a combination of two factors: the penetration depth of the initial projectile into the liquid, and the probability of the reporter species escaping from a given depth and being detected. The reporter may be the initial projectile itself (e.g., RBS, LEIS) or some secondary species that is generated by it (e.g., SIMS, NICISS, ARXPS, MAES/MIES, and RAS as used here).

The MAES/MIES methods, for example, achieve their high surface sensitivity through the very shallow penetration by the incident metastable He* atoms. However, this is not a requirement in RAS, nor the other related methods, if escape of the reporter is either prevented from greater depths, or its properties provide information on the depth from which it has been emitted. In ARXPS, as a contrasting example, the penetration of the initial ionizing X-ray photons into the bulk is very deep but the high probability of inelastic scattering allows elastic photoelectrons that were generated nearest the surface to be distinguished from those that originated at greater depths.

Surface sensitivity can also be enhanced by using near-grazing incidence angles, such as in SIMS. In its application to typical ILs, a high-energy ion projectile (e.g., He^+^ or C^+^) generates a wide range of secondary ions from species present at the surface.^30,38^ There have also been ion-scattering experiments at considerably lower incident energies, which are conceptually closely related to RAS and are known as ‘reactive-ion scattering’ (RIS).^57–59^ They have been applied to self-assembled monolayers (SAMs), which share many of the features of liquid surfaces. As a specific example, Jacobs and co-workers employed ∼5–40 eV incident O^+^ ions in combination with detection of product OH^–^ from thioalkyl SAM surfaces.^60,61^ This again exemplifies how surface sensitivity can arise through some combination of the penetration between the chains by the O^+^ projectile and successful escape of the OH^–^ reporter that is produced through chemical reaction and double electron transfer.

A particular feature of RAS-LIF, as previously implemented, was that it used photolytically generated O atoms at only moderately superthermal incident energies, with mean incident energy ⟨*E*_i_⟩ = 16 kJ mol^–1^.^45,46,48,49^ The dynamical characteristics of the product OH confirmed that they could not have suffered significant interactions with surrounding molecules before escaping into the gas phase. We have noted previously that the parallel RAS-MS measurements with higher-energy O atoms (⟨*E*_i_⟩ = ∼500 kJ mol^–1^) from a laser-detonation source gave qualitatively similar, but quantitatively different, results on surface composition from RAS-LIF.^49^ This implies that the degree of surface sensitivity is affected by the incident energy, with higher-energy projectiles being somewhat less sensitive to subtle differences in the surface structure.

The RAS-LIF results with low-energy O atoms led us to conclude that this technique approached a very extreme version of surface specificity, which we define to be detecting only those surface atoms that are impacted directly by the probe projectile approaching from the gas phase without encountering any other atoms. We denote this as ‘outer-surface exposure’. Part of its utility is that it is a limiting case that is rigorously defined and can be quantified through molecular dynamics simulations of the surface structure.^49,53,54,56^ The other limit would correspond to no surface sensitivity, where the observations of relative proportions of different chemical components should obviously converge on those of the bulk liquid. As we shall consider in detail for some examples in this work and is well established through extensive molecular dynamics (MD) simulations on a range of IL systems,^8,9,21,53,62,63^ the bulk structure is typically only fully developed at depths that correspond to several molecular-level layers. This whole ‘nonbulk’ region constitutes the surface in one sense, but the composition that is reported will depend on the specific range of depths that is sampled. Consequently, it is common for the apparent surface exposures of particular species to differ according to the techniques used.^30^

The successful measurements of alkyl-chain exposure provide the base from which we hope to expand RAS-LIF to other functional groups present at the surfaces of ILs. Specifically, in this work, we further explore a potential new RAS probe for the presence of *fluorinated* species at IL surfaces that we have introduced most recently.^64^ The desire to detect F is motivated both by its presence in a range of common anions used in ILs and, more specifically, is stimulated by our previous work on IL mixtures contain alkyl and fluoroalkyl chains.^56,65^ Through O atom RAS-LIF measurements, we were only able to infer preferential occupation of the surface by fluoroalkyl chains *indirectly*, on account of a deficit in the OH signal attributed to a reduction in exposed alkyl chains.^56^ It is, therefore, desirable in such circumstances to be able to confirm the surface fluorine by direct detection.

The interest in mixtures such as these, consisting of contrasting functionalities, is driven by the search for ILs that are optimized for particular applications. In general, trial-and-error testing of the very large number of conceivable binary combinations of cations and anions is both inefficient and practically impossible. A much more efficient strategy to fine-tune the resulting properties is, therefore, to prepare mixtures of ILs, such as those containing fluoroalkyl and alkyl substituents, in different proportions.^56,66–77^ The well-known differences in molecular-level properties (volume, stiffness, polarity, and polarizability) between alkyl and fluoroalkyl chains can be expected to lead to controllable changes in bulk and surface properties such as density, viscosity, hydrophobicity, surface tension, etc.

This work builds on our initial report that fluorinated surface groups can be detected via their reaction with an ablated Al plume.^64^ It was demonstrated that a pair of ILs containing the fluorinated bis­(trifluoromethylsulfonyl)­imide anion, denoted [Tf_2_N]^−^, successfully produced gas-phase AlF. The relative yields were consistent with previous LEIS measurements by Villar-Garcia *et al.*, which are one of the few existing sources of information on exposure of anions at IL surfaces.^32^ Although the primary motivation here is interest in developing new analytical probes of liquid surfaces, we note in passing that there are other contexts in which the reactions of energetic metallic species, and Al in particular, with the surfaces of liquids and other materials are important. These include energetic materials ignited by laser ablation, including Al in fluorinated matrices designed to be used as propellants,^78–80^ and the interactions of ablated Al with a liquid medium as a method for the synthesis of nanoparticles.^81^

RAS-LIF based on Al ablation and AlF detection is still in its infancy. It is not yet known which reactive species in the Al plume are responsible for AlF production, nor what effect the kinetic energies of those species might have on its surface specificity or generality for detection of different F-containing species. Indeed, AlF production is currently only established for one type of fluorinated anion, [Tf_2_N]^−^, as noted above.^64^ We extend that here by applying it to a broader range of ILs containing different fluorinated anions, including two fluorosulfonate anions, [Tf_2_N]^−^, as previously, and trifluoromethanesulfonate, [OTf]^−^, and the smaller inorganic tetrafluoroborate anion, [BF_4_]^−^. The F-atom chemical environments appear at least superficially similar in [Tf_2_N]^−^ and [OTf]^−^, but more obviously distinct in [BF_4_]^−^ (see Figure 1). We combine these three anions with two cations ([C_2_mim]^+^ and [C_8_mim]^+^ - Figure 1) from the well-studied 1-alkyl-3-methylimidazolium family, to assess any effect of alkyl chain length on detected levels of AlF.

**Figure 1:**
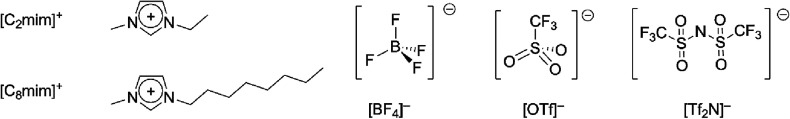
Chemical structures of the ionic components of the ionic liquids investigated in this work; [C_2_mim]^+^ = 1-ethyl-3-methylimidazolium, [C_8_mim]^+^ = 1-octyl-3-methylimidazolium, [Tf_2_N]^−^ = bis­(trifluoromethylsulfonyl)­imide, [OTf]^−^ = trifluoromethanesulfonate, [BF_4_]^−^ = tetrafluoroborate.

The resulting AlF yield measurements are compared with quantitative analysis of molecular dynamics (MD) simulations for all the studied ILs. Their outer surfaces are analyzed using an implementation of the ‘ball-drop’ method, conceptually similar to that first proposed by Pártay et al.,^82^ in combination with the well-established solvent-accessible-surface area (SASA) method.^83^ In this way, a quantitative measure of outer-surface exposure (as defined above) is obtained. The extent to which relative AlF yields reflect these predicted exposures is established, providing insight into the degree of surface sensitivity and scope of this new variant of the RAS-LIF method.

## Experimental Methods

Experiments were performed using the apparatus described previously,^64^ with two modifications. First, the ablation laser was upgraded from a Continuum Minilite II to a Continuum Surelite I-10, which has a higher pulse energy (and larger beam diameter), which allows a wider range of fluences to be investigated. Second, the apertures between the source and the wheel were reduced in size from their original diameters of 18 to 5 mm and 10 mm, respectively, for those closest to the source and to the liquid-coated wheel, to further ensure a well-collimated incident beam.

In brief, the second-harmonic (532 nm) output of a Nd:YAG laser was focused onto an Al-metal rod. The rod was rotated every fifth laser shot and translated vertically after a near-complete rotation by stepper motors to ensure consistency of production. The Al plume produced traveled 480 mm to the target liquid surface, passing through the two apertures on the way. Continually refreshed liquid surfaces were prepared on the surfaces of rotating stainless-steel wheels. Four equivalent wheels were mounted in a rotatable square assembly which allowed signals from up to four liquids to be compared directly without breaking vacuum.^51^ The gas-phase AlF generated was probed 10 mm in front of the wheel by laser-induced fluorescence (LIF). The probe-laser light was produced by frequency doubling the fundamental output of a Nd:YAG-laser-pumped dye laser, with an energy of 0.25 μJ in a ∼5 ns pulse at around 227 nm. The fluorescence was collected by a system of lenses and detected using a photomultiplier tube. The output signal was recorded using an oscilloscope.

The LIF signals were excited from the electronic ground state AlF on the A^1^Π-X^1^Σ^+^ transition. For the purposes of accumulating excitation spectra or appearance profiles, the LIF signal was isolated from residual scattered probe laser light in software by integrating over a time gate of width 15 ns immediately after the probe pulse. A background gate (width 60 ns) positioned before the probe pulse was used to correct for any DC fluctuations. For appearance profiles, signals were averaged over 50 laser shots per delay between ablation-laser and probe-laser pulses. Delays were chosen in a random order to minimize any systematic drift in source production or laser stability. Five individual profiles were recorded for one sample of liquid, immediately followed by five profiles of the reference liquid, [C_2_mim]­[Tf_2_N]. Each of these measurements was repeated at least three times independently on different days.

The ILs [C_2_mim]­[BF_4_], [C_8_mim]­[BF_4_], [C_2_mim]­[OTf], and [C_8_mim]­[OTf], were sourced commercially (io-li-tec, Germany). [C_2_mim]­[Tf_2_N] and [C_8_mim]­[Tf_2_N] were synthesized as described in our previous work.^76,84^ In the particular case of [C_2_mim]­[OTf], for reasons that will become clear below, we also synthesized a sample independently according to the method in the Supporting Information. We confirmed the chemical composition of both samples of [C_2_mim]­[OTf] using ^1^H and ^19^F NMR spectroscopy, as also described in the Supporting Information.

All samples were degassed in a separate purpose-built vacuum chamber at a pressure of <10^–6^ mbar for at least 3 h prior to being transferred to the reaction chamber, where they were held at a common temperature of 320 K and at a typical base pressure of <10^–7^ mbar for a period of at least 12 h before measurements were recorded.

## Computational Methods

### MD Simulation Procedure

MD simulations of the ILs were performed using GROMACS version 2022.1 and the CL&P extensions^85–89^ to the OPLS-AA force field.^90^ Simulations were performed for all systems consisting of 800 ion pairs following the procedure defined previously as ‘Protocol 1’,^56^ which we summarize here. First, the bulk liquid was simulated. Ions in a single conformation for each type were packed randomly into a cubic box of side length between 7 and 9 nm. Steepest-decent energy minimization was performed before each liquid was simulated under NPT conditions for ∼0.5 ns using a Berendsen barostat (1 bar) and velocity-rescaling thermostat at 500 K. Subsequently, the systems were simulated using a Parrinello–Rahman barostat (1 bar) and a velocity-rescaling thermostat at 320 K (the experimental temperature used in this work). The number densities of different atom types in the bulk phase were captured for comparison later in the analysis. The final frame from the bulk run was extended in the *z*-dimension by a factor of 3. This allowed the simulation of a slab approximately 7–9 nm thick with two vacuum interfaces while still using periodic boundary conditions. Under NVT conditions, 8 repeated cycles of 5 ns at 320 K followed by 5 ns at 500 K were performed to equilibrate the slab, before a final 10 ns simulation at 320 K was run. Results reported here were taken from averages over this final 10 ns run.

### MD Surface Analysis

Initially, the SASA algorithm^83^ incorporated in GROMACS was used to determine the areas of the fluorine atoms exposed at the surface. Following the procedure established in our O atom work,^56^ we used a probe-particle radius of 0.18 nm that is close to the van der Waals radius of an Al atom. Previously we had found that the SASA algorithm could identify unwanted contributions from voids in the bulk. It is possible to use thresholding approaches to suppress these contributions,^56^ but in this work we have developed an alternative ball-drop algorithm to identify unambiguously only surface atoms. This was an original implementation of conceptually similar approaches devised originally for water but also applied to IL surfaces, sometimes known as ‘identification of the truly interfacial molecules’ (ITIM).^82,91^ It is based on ‘dropping’ a probe-particle ball (again, here, of radius 0.18 nm) toward the surface along the surface normal (*z*-axis) with fixed *x* and *y* coordinates. The first atom which the ball hits (defined as a touching or overlap of the probe particle with the van der Waals volume of the atom in the simulation) is identified as a surface atom. This process is repeated over a mesh of spacing 0.02 nm in both *x* and *y* directions for both vacuum-liquid interfaces to identify all the surface atoms in the system. We note that, in general, for nonzero angles of incidence, balls should be dropped along vectors tracing out a cone around the surface normal with its tip at the point of impact to represent the different accessible pathways to hit an atom. However, in the current work we are using this approach to make comparisons with our experimental data for which experimental geometric constraints limit the maximum angle of incidence to < ±1°, so it is not necessary in this case.

Data from the ball drop and SASA approaches were combined to ensure that only the areas of atoms identified as being at the surface were included. The accessible areas of each of the target atoms were obtained from the SASA data, and the ball drop method was used to filter only those atoms present at the surface. The total area of all such atoms of each type present in a liquid was summed, allowing the fractional surface coverages by each atom type to be determined. This analysis was performed on selected frames from the MD trajectory spaced 0.4 ns apart; this value was determined by a block-analysis approach,^92^ ensuring that only broadly uncorrelated frames are analyzed.^56^

Z-density profiles (number density as a function of distance along the surface normal) for different atom types were determined by averaging over snapshots at the same set of intervals, using the standard tools available within GROMACS.

## Results

### RAS-LIF Measurements

As in our previous work,^64^ the identity of the species detected following exposure of the IL surfaces to the Al-ablation plume was confirmed to be AlF by recording LIF excitation spectra, as in the representative example in Figure 2. This shows clearly the expected structure, as evident from the simulation using the PGOPHER package,^93–96^ of the AlF­(A^1^Π-X^1^Σ^+^) diagonal bands, and, in addition, contains some information about the rovibrational distribution of the AlF escaping the surface. Note that although an assumed rotational temperature of 300 K matches the experimental rotational distribution reasonably well, it is necessary to include small but significant populations in vibrational levels up to at least *v*′ = 5 to reproduce the observed weaker bandheads on the higher diagonal bands.

**Figure 2:**
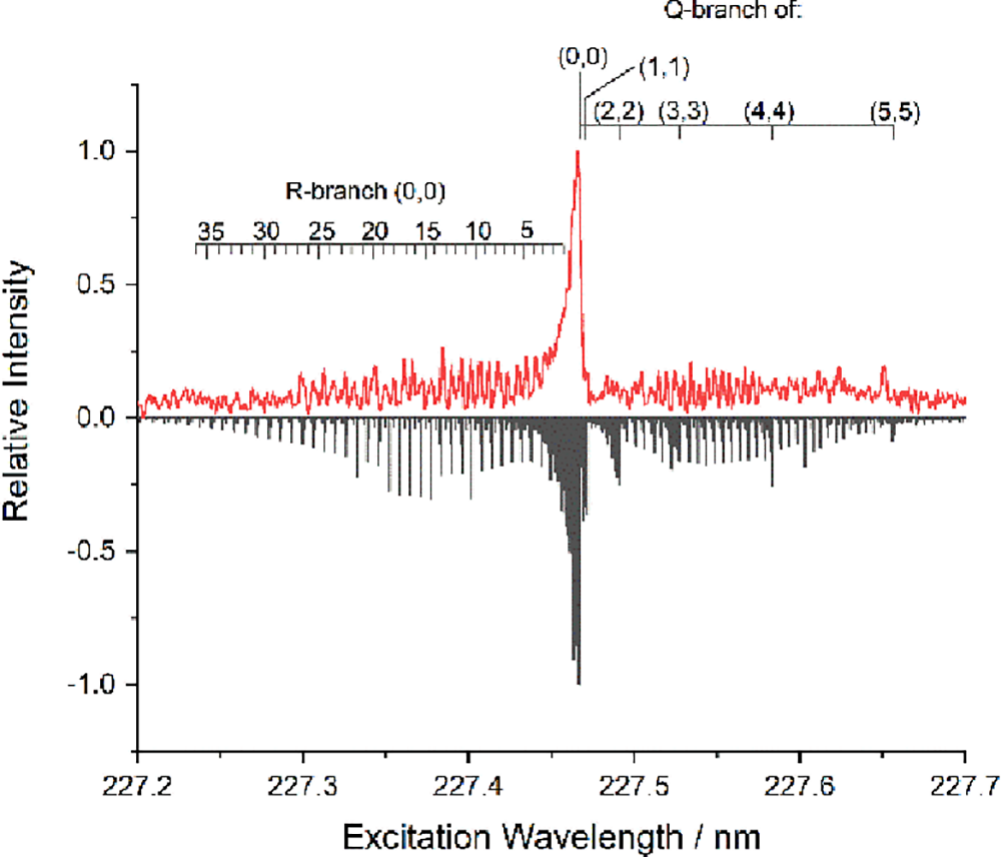
LIF excitation spectrum (upper trace - red line) of AlF­(A^1^Π-X^1^Σ^+^) diagonal bands recorded from reaction of the Al plume with [C_2_mim]­[Tf_2_N]. Recorded at an ablation-probe delay of 50 μs, with 50 shots per point. Comparison with PGOPHER^93–96^ simulation (lower trace – black line), assuming a rotational temperature of 300 K for all vibrational levels and a synthesis of several vibrational temperatures up to 1500 K.

AlF appearance profiles (see Figure 3) were recorded on the peak of the dominant Q-branch bandhead of the A-X (0,0) band at 227.4 nm for the full range of ILs and at two different ablation-laser fluences, which we denote ‘low’ (11.3 J cm^–2^; corresponding to a 8.0 mJ pulse in a nominal 7.07 × 10^–4^ cm^2^ area) and ‘high’ (42.4 J cm^–2^; 30.0 mJ pulse in the same area) fluence. For comparison, the empirical threshold for generating ablated species that results in measurable AlF yields is around 3 mJ pulse^–1^ (or 4 J cm^–2^) with our current experimental arrangement. AlF signals in Figure 3 are normalized to those from [C_2_mim]­[Tf_2_N] at high fluence.

**Figure 3:**
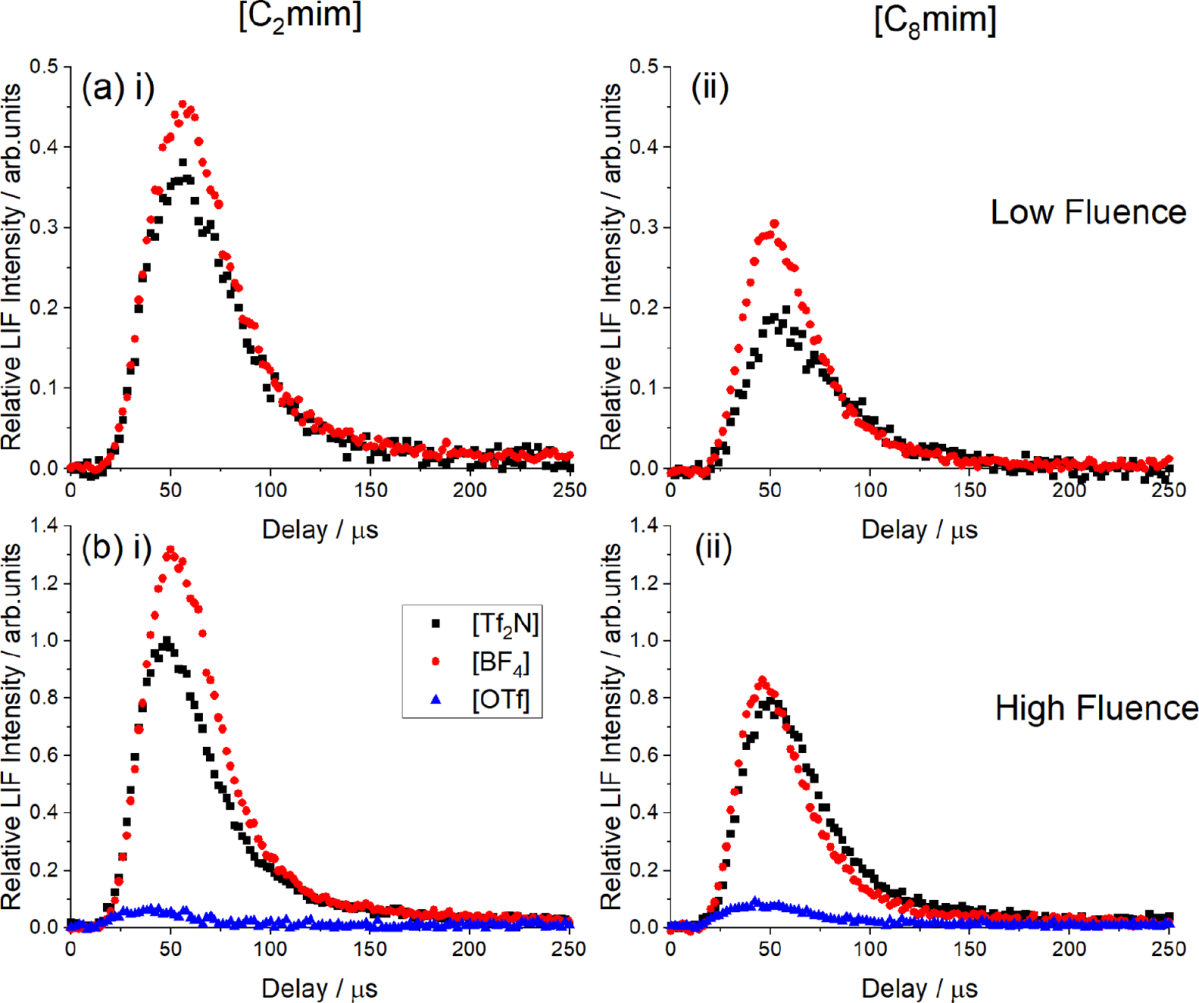
Appearance profiles of AlF recorded at (a) low and (b) high ablation fluence for (i) [C_2_mim]­[X] and (ii) [C_8_mim]­[X], where [X] = [Tf_2_N] (black), [BF_4_] (red) and [OTf] (blue). Signals are normalized to that of [C_2_mim]­[Tf_2_N] at high fluence. The intensity scales in (a) and (b) correctly reflect the measured low-fluence-to-high-fluence ratio of signals for [C_2_mim]­[Tf_2_N] of 0.36 ± 0.01.

As Figure 3b shows, at the high ablation fluence clear AlF signals were detected from ILs containing all three fluorinated anions, yet the AlF yields vary substantially, with relative intensities from [C_2_mim]^+^ ILs in the sequence [BF_4_]^−^ > [Tf_2_N]^−^ ≫ [OTf]^−^. This sequence is essentially retained for [C_8_mim]^+^ ILs, but with overall reductions in yield relative to [C_2_mim]^+^ for both the [BF_4_]^−^ and [Tf_2_N]^−^ salts. The yield from [C_8_mim]­[OTf] remains much lower than from the other two [C_8_mim]^+^ ILs, although if anything has increased marginally over that observed for [C_2_mim]­[OTf]. These low yields from [OTf]^−^-containing ILs were a considerable surprise, motivating us to confirm them in separate experiments using independent sources of [C_2_mim]­[OTf] , as shown in the Supporting Information. We conclude, therefore, that the low yields are an authentic feature of these [OTf]^−^ salts.

AlF yields were, not surprisingly, generally suppressed at lower Al-ablation fluences. The estimated relative yield of [C_2_mim]­[Tf_2_N] at low fluence is (36 ± 1)% of that at high fluence (hence note that this is reflected in the scales in Figure 3a,b). Inspection of Figure 3 shows that there are only quite subtle effects of fluence on the relative yields from salts with [Tf_2_N]^−^ and [BF_4_]^−^ anions, which also depend only moderately on alkyl chain length of the cation. The already-weak signals from [OTf]^−^ became too small at these lower ablation fluences to be quantified reliably and have, therefore, been omitted.

Quantitative yields of AlF were derived by integrating the appearance profiles between delays of 20 and 150 μs, capturing the majority of the signals for all the ILs. (The upper limit is set to avoid any contribution from AlF molecules that may have suffered secondary collisions with surrounding parts of the apparatus and returned to the probe region.) The data are summarized in Table 1. The one previous result with which we can compare directly is our own proof-of-concept measurement of 0.52 ± 0.05 for the relative AlF yields from [C_8_mim]­[Tf_2_N] and­[C_2_mim]­[Tf_2_N].^64^ This was taken under relatively low-fluence conditions, with a pulse energy of 8 mJ. This is nominally the same as our low-fluence measurements here, which give a ratio of 0.586 ± 0.002. This level of agreement, almost within the 1σ uncertainties, is considered reasonably good, given the potential differences in the ablation-laser beam profiles and that the results here vary with fluence, increasing to 0.821 ± 0.036 at the higher pulse energy of 30 mJ.

**Table 1:** Measured Relative RAS-LIF Yields of AlF at Different Ablation Fluences and MD Predictions of Relative F-Atom Outer-Surface Exposure in Different ILs^a^

	RAS-LIF AlF yield		MD F-atom outer-surface exposure	
ionic liquid	low fluence rel to ref^b^	high fluence rel to ref^c^	rel to ref^d^	absolute fraction^e^
[C_2_mim][Tf_2_N]	1	1	1	0.605 ± 0.002
	low:high^g^ = 0.36 ± 0.01			
[C_8_mim][Tf_2_N]	0.586 ± 0.002	0.821 ± 0.036	0.274 ± 0.004	0.166 ± 0.003
	low:high^g^ = 0.30 ± 0.02			
C_8_/C_2_ [Tf_2_N]^h^	0.586 ± 0.002	0.821 ± 0.036	0.274 ± 0.004	
[C_2_mim][BF_4_]	1.170 ± 0.026	1.266 ± 0.026	0.339 ± 0.004	0.205 ± 0.002
	low:high^g^ = 0.33 ± 0.02			
[C_8_mim][ BF_4_]	0.787 ± 0.052	0.774 ± 0.038	0.046 ± 0.001	0.028 ± 0.001
	low:high^g^ = 0.37 ± 0.02			
C_8_/C_2_ [ BF_4_]^i^	0.673 ± 0.047	0.611 ± 0.033	0.137 ± 0.004	
[C_2_mim][OTf]		0.067 ± 0.004^j^	0.868 ± 0.005	0.525 ± 0.003
[C_8_mim][OTf]		0.085 ± 0.012	0.134 ± 0.003	0.081 ± 0.002
C_8_/C_2_ [OTf]^f^		1.27 ± 0.19	0.154 ± 0.003	

a Quoted uncertainties are 1σ standard error in the mean, based on repeated measurements of integrated appearance profiles (RAS-LIF yields) or variations between multiple independent equilibrated snapshots as described in the text (MD).

b AlF yield relative to reference material, [C_2_mim]­[Tf_2_N], at an ablation pulse energy of 8 mJ.

c AlF yield relative to reference material, [C_2_mim]­[Tf_2_N], at an ablation pulse energy of 30 mJ.

d MD-predicted ratio of absolute fractional coverage to that of the reference material, [C_2_mim]­[Tf_2_N].

e MD-predicted fraction of the surface area occupied by F atoms, as defined in the text.

f Ratio of low-fluence to high-fluence AlF yields for each IL.

g Ratio of AlF yields from [C_8_mim]­[Tf_2_N] and [C_2_mim]­[Tf_2_N]

h Ratio of AlF yields from [C_8_mim]­[ BF_4_] and [C_2_mim]­[ BF_4_]

i Average of the commercial and self-synthesized [C_2_mim]­[OTf] samples.

j Ratio of AlF yields from [C_8_mim]­[OTf] and [C_2_mim]­[OTf]

### MD Simulations

MD simulations were carried out for all six salts to gain a better understanding of the nature and organization at the IL surfaces. Representative snapshots from the fully equilibrated sections of the MD trajectories are shown in Figure 4 (plan view) and Figure 5 (side view). Color coding is designed to emphasize exposure of F atoms; those shown in blue are accessible to the probe particle in the ball-drop method. Those in pink are visible by eye to an observer looking vertically down on the surface, but not accessible to the probe particle with the chosen radius due to the proximity of other groups.

**Figure 4:**
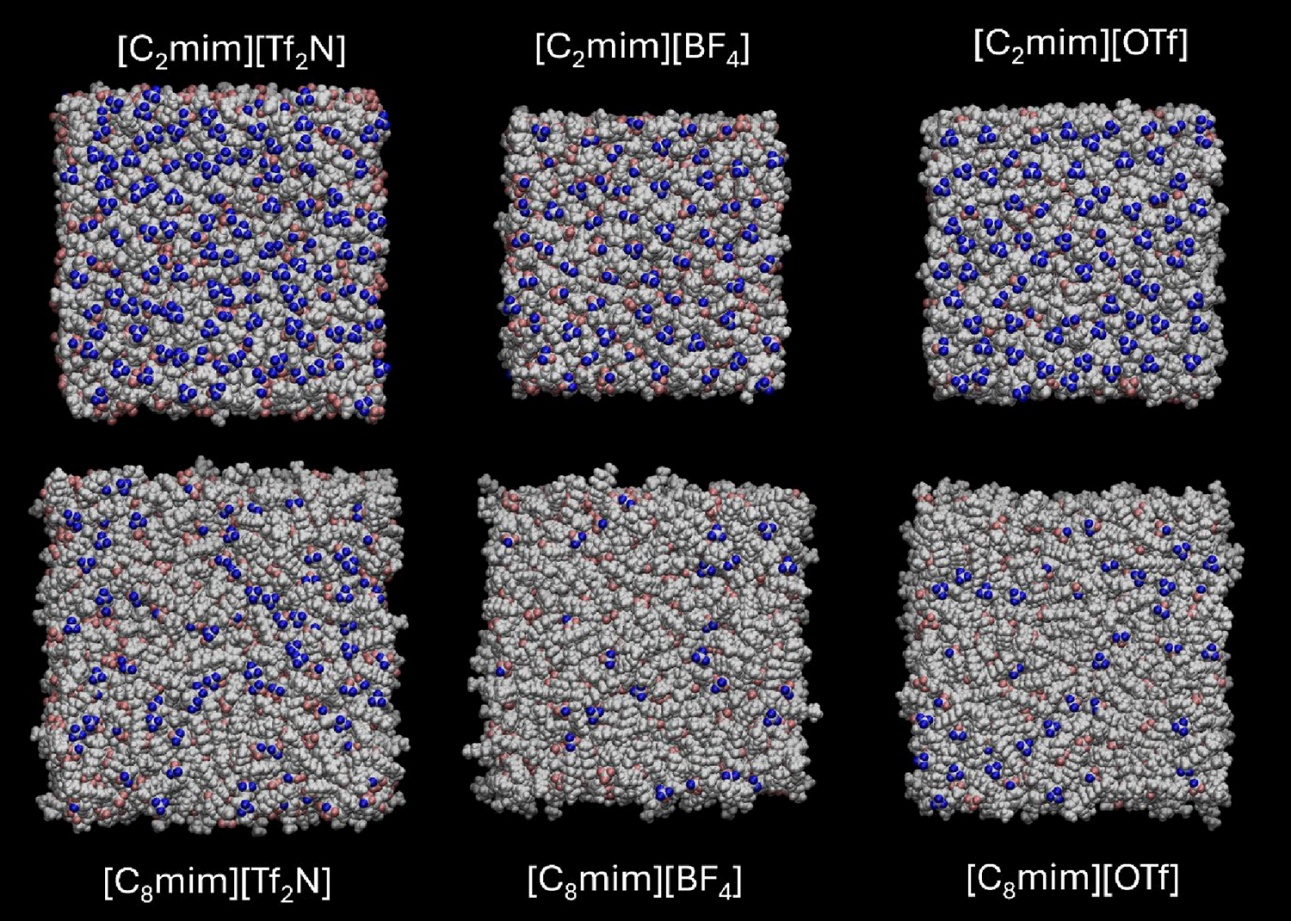
Top-down views of the final frame of MD simulations for each combination of cation and anion, as indicated. Fluorine atoms identified by the ball-drop method as being accessible are shown in blue; fluorine atoms visible by eye but not identified by the ball-drop method are shown in pink; all other atoms are shown in white.

**Figure 5:**
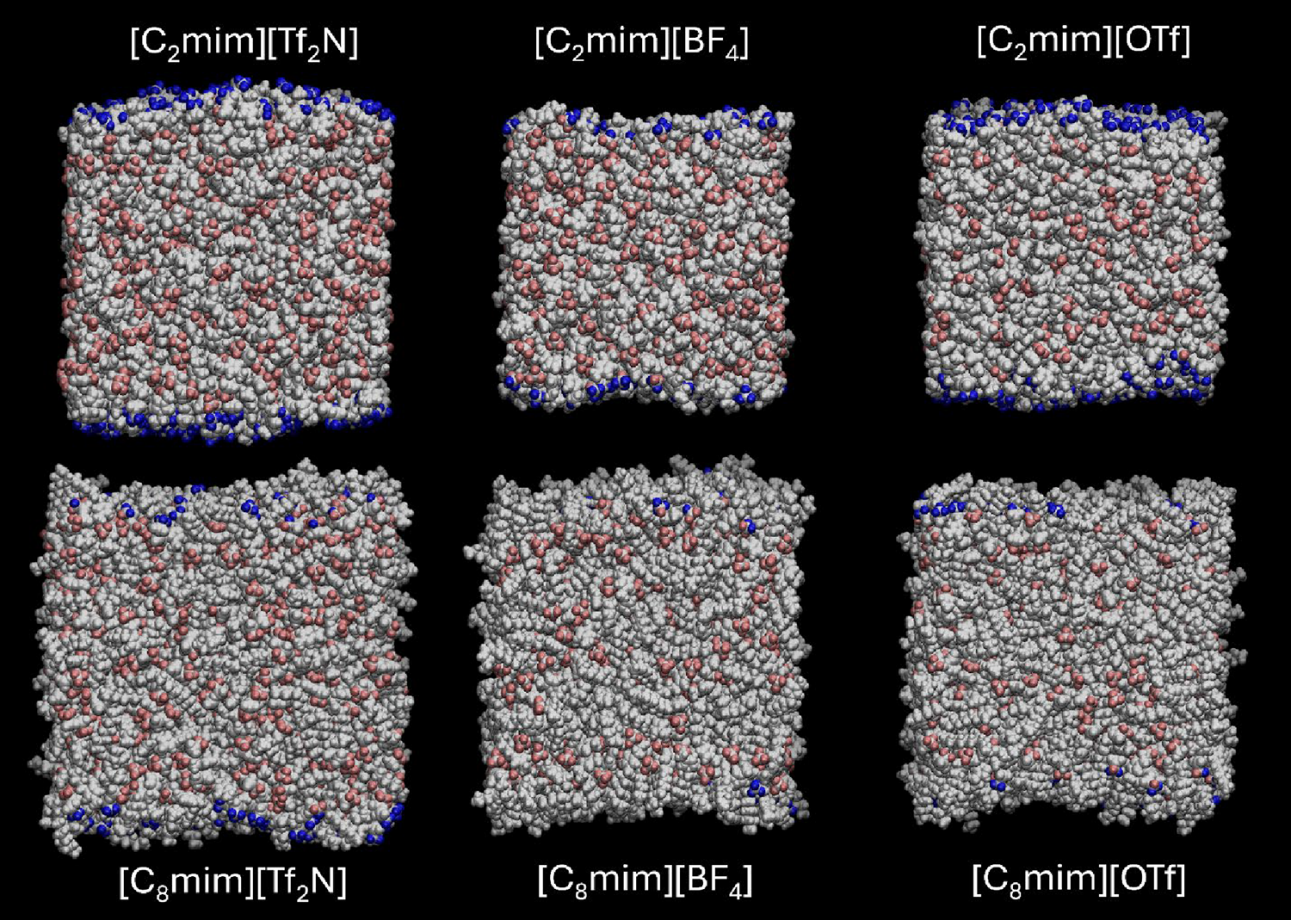
Side-on views of the final frame of MD simulations for each combination of cation and anion, as indicated. The scheme for labeling of atoms is the same as in Figure 4.

Inspection of Figure 4 suggests that the ratio of exposed F atoms per unit area for the [C_2_mim]^+^ ILs is in the sequence [Tf_2_N]^−^ > [OTf]^−^ > [BF_4_]^−^. This sequence is maintained for [C_8_mim]^+^ ILs, but with a very substantial reduction in F-atom exposure for all three anions, with the surfaces unsurprisingly becoming visibly more heavily dominated by the longer alkyl chains. This also leads to a general increase in molecular-scale roughness, as can be seen in Figure 5.

These qualitative observations were confirmed by the quantitative analysis of the outer-surface exposure (as defined above) of atoms of different types. Results were averaged over a total of 23 snapshots (spaced by 0.4 ns to ensure they were effectively uncorrelated, as explained above) over the final 20 ns of the trajectories in the fully equilibrated region. As described above, a combination of SASA and ball-drop analyses were carried out and, as we show in the Supporting Information, there are only marginal differences in the F atoms identified as being exposed. The results quoted in Table 1 are derived from the exposed areas, as determined by SASA, for atoms which also passed the ball-drop test. Two quantities are listed in Table 1. One is the absolute fraction of the exposed surface area that consists of F atoms; this normalizes out trivial differences in simulation-box dimensions for different ILs and different total surfaces areas, which reflect the general increase in roughness in going from [C_2_mim]^+^ to [C_8_mim]^+^ salts, noted above. The absolute fraction produces a measure proportional to the probability of an F atom, as opposed to any other atom-type, being struck as the probe particle first encounters the surface. This corresponds to our definition of ‘outer-surface exposure’, introduced earlier. The other quantity in Table 1 is the ratio of this F-atom fraction to that for the reference IL, [C_2_mim]­[Tf_2_N], which can be compared directly with the RAS-LIF AlF yields from different ILs.

Separately, we derived the bulk number densities of different atom types from the bulk simulation that preceded the expansion of the box to form the liquid slab (see above). These are needed for comparison in the discussion that follows and are presented below.

## Discussion

We consider first the aspects of the new RAS-LIF observations that fit qualitatively with expectations or corroborate previous quantitative results. ILs containing either [Tf_2_N]^−^ or [BF_4_]^–^ show the expected qualitative trend with alkyl chain length, with the AlF yield being lower for [C_8_mim]^+^ than [C_2_mim]^+^-containing ILs. The quantitative ratios depend on fluence for [Tf_2_N]^−^, increasing from 0.586 ± 0.002 at low fluence to 0.821 ± 0.036 at high fluence. As noted above, the low-fluence result is close to our own previous value of 0.52 ± 0.05 using the same RAS-LIF method at a nominally similar fluence.^64^ It is independently corroborated by LEIS measurements by Villar-Garcia et al., which gave a ratio of F-atom peaks of 0.52 ± 0.07.^32^ For [BF_4_]^–^, the results are very similar at both fluences, 0.79 ± 0.05 and 0.77 ± 0.04, respectively. We are not aware of any previous comparative measures of F-atom exposures for these two liquids.

At a qualitative level, these results confirm that the choice of fluorinated anion and variation of the cation alkyl chain length do provide a method for tailoring the composition of the IL surface, and hence a potential route to altering their performance in e.g. catalytic applications, as discussed in the Introduction. Interestingly, though, neither of these measured [C_8_mim]^+^/[C_2_mim]^+^ ratios is predicted accurately by the MD simulations. The predicted outer-surface exposures do decline from [C_2_mim]^+^ to [C_8_mim]^+^, as widely accepted to be the result of the longer alkyl chain occupying a larger fraction of the surface and hence obscuring the anions. However, the magnitude of the decline is considerably larger than the observations for [Tf_2_N]^−^, with a [C_8_mim]^+^/[C_2_mim]^+^ F-atom exposure ratio of 0.27. The predicted decline is even more extreme for [BF_4_]^–^, with a [C_8_mim]^+^/[C_2_mim]^+^ ratio of only 0.14. To allow the level of these disagreements to be assessed visually, the RAS-LIF results are compared graphically with the MD-predicted outer-surface exposures in Figure 6.

**Figure 6:**
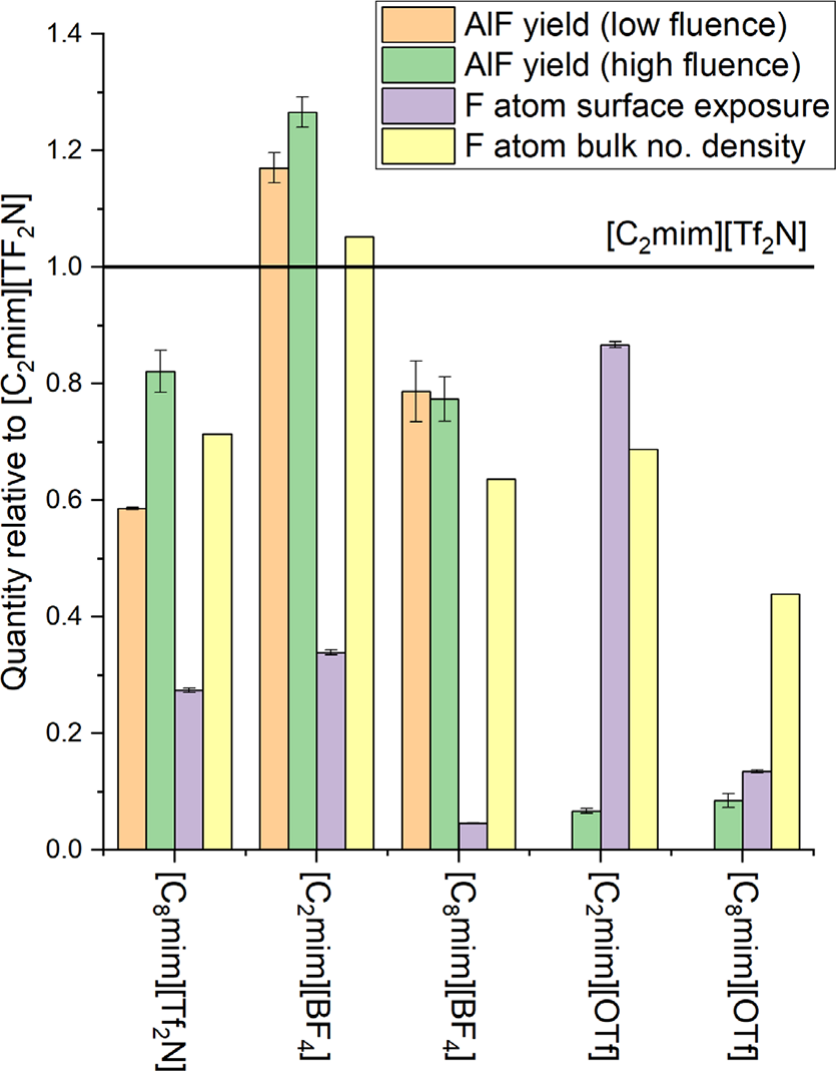
Relative RAS-LIF AlF yields at low ablation fluence (orange); high ablation fluence (green). MD predictions of relative F-atom outer-surface exposure (purple); MD predictions of relative F-atom bulk density (yellow). In all cases, data are normalized relative to the same measure for [C_2_mim]­[Tf_2_N].

Further disagreements are found between predicted outer-surface exposures and AlF yields for ILs containing [Tf_2_N]^−^ and [BF_4_]^–^ with a common cation. The MD-predicted ratio for [C_2_mim]­[BF_4_] versus [C_2_mim]­[Tf_2_N] is 0.34; the corresponding ratio of AlF yields varies slightly with fluence, from 1.17 to 1.27, but is clearly much larger. Likewise, for [C_8_mim]­[BF_4_] versus [C_8_mim]­[Tf_2_N], MD predicts a ratio of 0.17 in contrast to 1.34 and 0.94 for low and high-fluence AlF yields, respectively. All these differences are well beyond the statistical uncertainties, as is clear from both Table 1 and Figure 6. This is perhaps not too surprising given the very different chemical environments of the F atoms in [Tf_2_N]^−^ and [BF_4_]^–^. In common with many of the alternative surface probes noted in the Introduction, RAS-LIF is therefore not a ‘universal’ surface F-atom probe with constant detection sensitivity independent of the nature of the F-containing species. Nevertheless, this does not prevent such methods from being useful for the measurement of relative exposures of the same target species in different materials.

However, perhaps the most striking and surprising observation is the very large difference in AlF yields (see Figure 3 and Table 1) between the ILs with [Tf_2_N]^−^ and [OTf]^−^ anions, despite the apparently similar chemical environments of the F atoms. This persists for both [C_2_mim]^+^ and [C_8_mim]^+^ salts. Some differences might, of course, be expected between [Tf_2_N]^−^ and [OTf]^–^ simply based on the number of F atoms per anion and perhaps also on a more detailed consideration of the electronic environment of the respective CF_3_ groups. More subtly, the average surface area occupied by each of the anions and its orientation at the interface can be expected to differ. However, these liquid-structural effects should be captured in the MD simulations (Figures 4 and 5) and their analysis to produce outer-surface exposures (Table 1), yet they are clearly substantially different from the AlF yields for these two anions, as illustrated in Figure 6. The MD simulations predict that the ratio of F-atom exposures in [C_2_mim]­[OTf] and [C_2_mim]­[Tf_2_N] is 0.87. This greatly overestimates the observed ratio of only 0.067 in the high-fluence AlF yields. A similar, only slightly less-extreme, trend is observed for [C_8_mim]­[OTf] versus [C_8_mim]­[Tf_2_N]; the MD predicted ratio of F-atom exposures is 0.49, whereas the ratio of AlF yields is 0.10. The [OTf]^−^-containing materials are further anomalous in producing a small but probably statistically significant *increase*, by a factor of 1.27 ± 0.19, in the AlF yield from [C_2_mim]­[OTf] to [C_8_mim]­[OTf]. The MD prediction is for a *decrease*, by a factor of 0.154, qualitatively consistent with the effect of cation alkyl chain length in [Tf_2_N]^−^ and [BF_4_]^–^.

The clear conclusion is that the RAS-LIF AlF yields are not a straightforward measure of the outer-surface F-atom exposures, as we are defining them here, even for the same F-containing anion in different ILs. It is not certain that the MD predictions are correct, yet despite the known limitations on physical accuracy of structures calculated using empirical force fields, we consider it unlikely that they are the principal causes of the substantial discrepancies with the observed AlF yields. It is, therefore, more likely that they reflect a combination of factors in the RAS-LIF method, of the types introduced above, including penetration depth of the Al probe projectiles; the probability that they react to produce the reporter species, AlF; and that the AlF survives and returns to the gas phase.

To assess the first of these factors, we consider the number density of different atom types as a function of distance along the normal through the interfaces of the different liquids – known widely as the ‘*z*-density’ – obtained from the MD calculations. These are illustrated for all six ILs in Figure 7. Consistent with previous similar simulations, the plots show the relatively modest surface layering in [C_2_mim]^+^ ILs compared to the [C_8_mim]^+^ ILs.^21,49,53,54,56,62,63^ The longer-chain ILs have much stronger primary oscillations in the interfacial region, corresponding to nonpolar and ionic layers, with secondary oscillations that propagate further into the bulk. (We have confirmed that the oscillations visible for several atom types for all the ILs are reproducible by analyzing an equivalent sequence of snapshots with an equal time spacing, but shifted by half the interval between them. In our previous work on one of the liquids here, [C_8_mim]­[Tf_2_N], we have also confirmed that the oscillations persist when the slab thickness is doubled.^56^) Fluorine atoms are relatively prevalent at the extreme outer surfaces of the [C_2_mim]^+^ salts, especially for [Tf_2_N]^−^ and [OTf]^−^. Consistent with Figure 4 and the surface-exposure analysis in Table 1, the anions are significantly overshadowed by components of the alkyl chain in the [C_8_mim]^+^ ILs.

**Figure 7:**
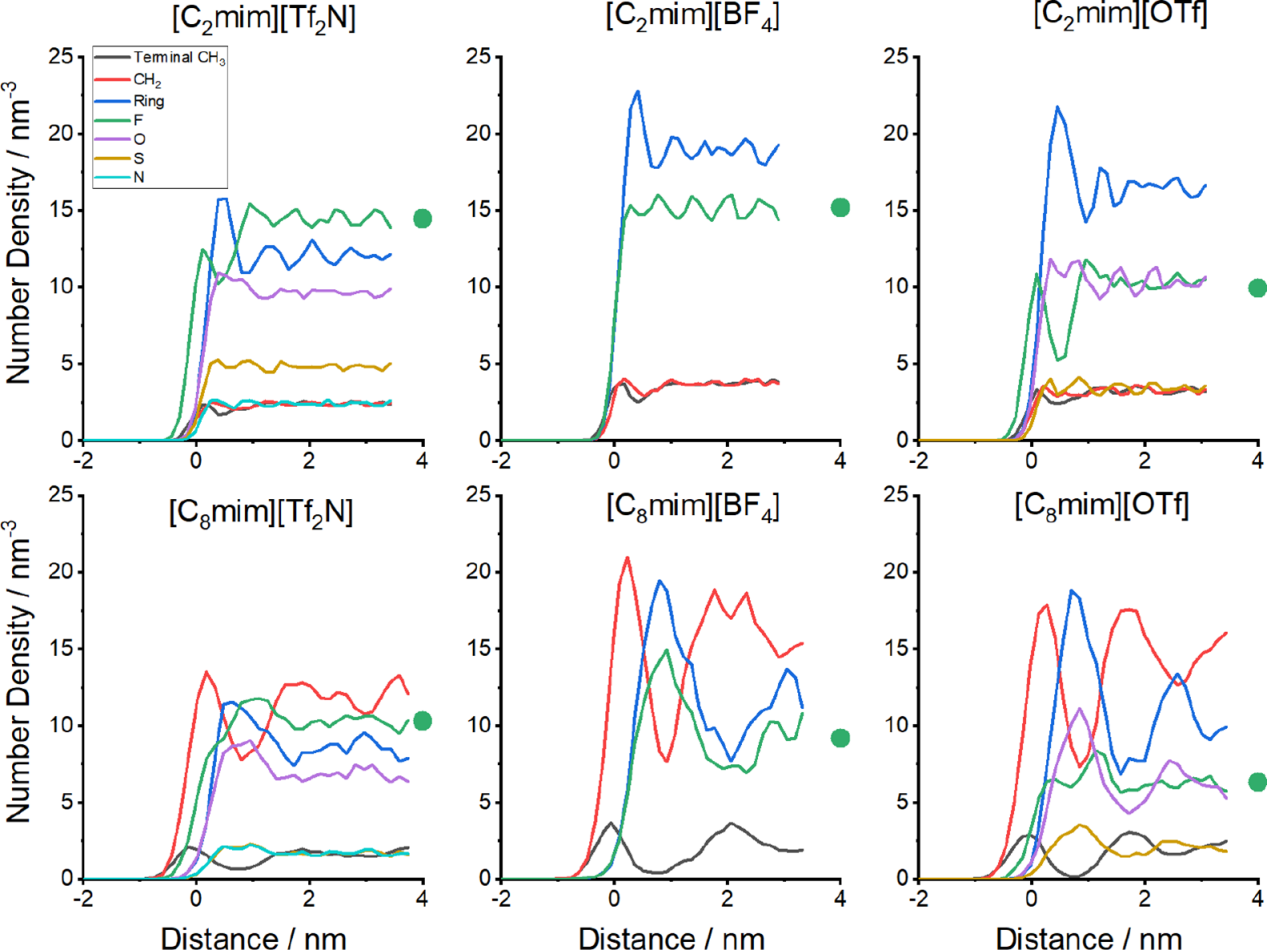
MD-predicted number densities of different atoms (F, O, S, N) or united atoms (CH_3_, CH_2_, ring-atom) as a function of distance along the surface normal (*z*-density) for each of the ILs, as indicated. Atom types are distinguished by the colors given in the legend. The zero of distance is defined as the midpoint between the 10% and 90% points of the total number density of all atoms. The filled green circle indicates the number density of F atoms in the bulk, determined separately in the initial stage of each MD simulation.

As noted above, in the limit that the effective probe depth extends significantly below the structured region of the surface, the relative F-atom abundances should approach their relative bulk number densities. The values calculated from the initial bulk phase of the simulation procedure have been added to Figure 7; they agree well with visual extrapolations of the surface simulations. The results relative to [C_2_mim]­[Tf_2_N] are included in Figure 6. In general, at least for the [Tf_2_N]^−^ and [BF_4_]^–^ salts, the relative bulk number densities are closer to the relative AlF yields than are the outer-surface exposures. This could imply that the Al projectiles penetrate significantly into the liquids, producing AlF which survives and escapes. This is perhaps not surprising given the known relatively high kinetic energies of the Al atoms; under our previous ablation conditions which correspond approximately to the low-fluence measurements here, the Al-atom speed distribution was approximately Maxwellian at ∼45,000 K, with a most-probable kinetic energy of 187 kJ mol^–1^ and a mean of 560 kJ mol^–1^_._ ^64^ Although, as noted above, O atom and F atom projectiles with comparable energies have been shown to have surface sensitivity in previous RAS-MS experiments,^47,49,50,54,55^ it is plausible that reactions of these relatively energetic species is not confined to the extreme outer layer as we define it here. Indeed, visual assessment of Figure 4 shows that only a relatively modest displacement of overlying alkyl chains would be necessary to provide access to additional fluorinated anions immediately below the surface. This would be even more likely for Al^+^ ions which, based on previous independent characterization of the laser-ablation process,^97^ may be present with even higher kinetic energies and could contribute to the observed AlF yields.

However, this does not necessarily amount to a conclusion that the Al-ablation probe is not at all surface sensitive; to the contrary, the relative AlF yields are ablation-fluence dependent, at least for [C_8_mim]­[Tf_2_N] versus [C_2_mim]­[Tf_2_N], with the low-fluence result being below the ratio of bulk densities and deviating in the direction of the MD-predicted outer-surface exposures. This is consistent with lower-energy projectiles penetrating less deeply, as expected. This could, nevertheless, be a coincidence given the other factors we consider shortly. Other circumstantial evidence in favor of a degree of surface sensitivity is the internal state distribution of the AlF. As shown in Figure 1, the AlF rotational distribution is close to thermal at the temperature of the liquid, which does suggest significant interactions with the surrounding molecules prior to escape. However, in contrast, the vibrational distribution is not fully thermalized, which puts some limit on residence time and hence average depth of formation.

These considerations of penetration by the probe may, therefore, go some way to rationalizing the relative yields for the [Tf_2_N]^−^ and [BF_4_]^–^ salts, but they still do not account for the very unexpectedly low relative yields for those containing [OTf]^−^. Indeed, there is nothing obvious in the *z*-density profiles for the [OTf]^−^-containing liquids in Figure 7 that suggests they have a qualitatively different structure from the other ILs. Likewise, there is also no suggestion from the side-on snapshots in Figure 5, or the absolute surface areas given in the Supporting Information, that they have an anomalous level of roughness. (We have confirmed that the selected snapshots shown in Figures 4 and 5 are typical of a wider range of samples.) Other factors, specifically either a lower probability of reaction to produce AlF or its failure to survive in the presence of [OTf]^−^ due to some efficient secondary reaction, must presumably be dominant. Given the high energies of the projectiles, there are a considerable number of thermodynamically open competing primary and secondary reaction channels for all of the ILs here. It is certainly known that a range of different charged products are produced in the RIS experiments of Pradeep *et al*. in studies of fluorinated SAM surfaces using primary ions, including Al^+^, that had similar kinetic energies to those that may be present here.^57^ The observed product ions include AlF^+^ and AlF_2_^+^, along with CF^+^, CF_3_^+^ and several heavier, singly charged fluorocarbon ions. The existence of competing primary and secondary reactions is not confined to [OTf]^−^, though, so it is an open question in which respect it differs significantly from [Tf_2_N]^−^ and [BF_4_]^–^. It is not obvious why secondary reactions of AlF with [OTf]^−^ should be more efficient than for the other anions, but one interesting possibility is that the more-strongly coordinating nature of [OTf]^−^ leads to efficient formation of AlOTf, which may compete effectively with primary AlF production. This would be more straightforward mechanistically if the reactive projectile was Al^+^, because no charge transfer would be required to produce a neutral product. It is likely that further work with a wider range of fluorinated anions, and indeed extending to the fluorine-containing cations that partially motivated this study, will be necessary to establish the factors that affect the relative yields of AlF from different fluorinated structure types. It would clearly be advantageous in this regard to further characterize the Al species present in the ablation plume and their potential contributions to the observed AlF yield. It would also be interesting to apply some of the available alternative techniques, particularly those believed to have high surface sensitivity such as MAES/MIES, LEIS or ARXPS, to obtain independent complementary information on surface structure in this class of ionic liquids.

## Conclusions

This study has demonstrated observation of AlF from a wider range of fluorinated anions in ionic liquids than investigated previously by RAS-LIF using an Al-ablation plume as the probe projectiles. Results obtained are compatible with those obtained from other surface-sensitive experiments where comparable data are available, showing the ability of the methodology to detect fluorinated species.

AlF yields are generally greater for salts of the shorter-chain cation [C_2_mim]^+^ compared to [C_8_mim]^+^, consistent with qualitative expectations of greater overshadowing of anions by longer alkyl chains. They are also slightly greater for [BF_4_]^−^ salts compared to those of [Tf_2_N]^−^. Surface availability of fluorine as determined from a combination of SASA and ball-drop approaches to surface analysis of MD simulations does not quantitatively reproduce the experimental relative AlF yields well. For these anions, better correlation with experiment is found for the calculated bulk F-atom number densities. This is consistent with significant penetration of the reactive projectile into the liquid.

However, no aspects of the MD simulations account for the very much lower yields of AlF from [OTf]^−^ salts, whose unexpected variation with alkyl chain length also cannot be rationalized by arguments based on penetration depth. This implies that they are dominated either by branching into other reaction channels which compete with AlF production, or by an efficient secondary loss process that removes AlF.

Despite these anomalies, which will require further investigations to rationalize, the work provides new physical insight into the reactions of energetic Al species with fluorinated compounds, which is of interest in other applications beyond surface analysis such as energetic materials used as propellants or explosives.

## Supplementary Material



## Notes

Synthesis and characterization of [C_2_mim]­[OTf]; RAS-LIF AlF yields from alternative samples of [C_2_mim]­[OTf]; and accessible surface areas from SASA and ball-drop methods (PDF)
