# Supplementary material for: Detection of Different Classes of Fluorinated Anions at Ionic-Liquid Surfaces by Reactive-Atom Scattering Using Laser-Ablated Al Projectiles
Source: J Phys Chem C Nanomater Interfaces. 2026 Feb 12;130(8):2975–87. doi: 10.1021/acs.jpcc.5c08388 (PMC12951565; doi:10.1021/acs.jpcc.5c08388)
Supplement: Supplementary file 1 [file jp5c08388_si_001.pdf]

# Detection of Different Classes of Fluorinated Anions at Ionic-Liquid Surfaces by Reactive-Atom Scattering using Laser-Ablated Al Projectiles

*Paul D. Lane,[1] Naomi S. Elstone,[2] Duncan W. Bruce,[2] John M. Slattery,[2] Matthew L. Costen,[1] and Kenneth G. McKendrick*[1]*

1. Institute of Chemical Sciences, School of Engineering and Physical Sciences, Heriot-Watt University, Edinburgh, EH14 4AS, UK

2. Department of Chemistry, University of York, Heslington, York YO10 5DD, UK

**Supporting Information**

Contents

S1. Synthesis and characterization of [$C_2$mim][OTf].

S2. RAS-LIF AlF yields from alternative samples of [$C_2$mim][OTf].

S3. Accessible surface areas from SASA and ball-drop methods.

## S1. Synthesis and characterization of [$C_2$mim][OTf]

[$C_2$mim][OTf] was synthesized by the following method. Under a $N_2$ atmosphere, 16.5 mL ethyl trifluoromethanesulfonate (freshly distilled) (0.127 mol) was added dropwise to distilled methylimidazole (9.8 mL, 0.123 mol) cooled in an ice bath at 0$^{o}$C. It is important to add the ethyl trifluoromethanesulfonate very slowly to avoid excessive heating, due to the exothermic reaction. A solvent, such as toluene, can be used to help control the reaction temperature and is important for larger-scale reactions. Once addition was complete the flask was allowed to return to room temperature and was stirred overnight in the dark. The product was dried under vacuum at room temperature for 2 days until no starting material signals were present in the $^{1}$H NMR spectra. The total yield was 31.47 g (98%).

We assessed the chemical composition of both the commercial and in-house prepared samples of [$C_2$mim][OTf] by $^{1}$H and $^{19}$F NMR spectroscopy, which confirmed that both were indeed [$C_2$mim][OTf]; the [OTf]$^{-}$ anion was present and no other fluorinated anions were observed. The commercial sample of [$C_2$mim][OTf] (45 mg, 0.173 mmol) was investigated by quantitative $^{19}$F NMR with an internal standard (2,3,4,5,6-pentafluorotoluene, 17 mg, 0.093 mmol), which confirmed the presence of 1 equivalent of [OTf]$^{-}$ for each [$C_2$mim][OTf] ion pair, as illustrated in Figure S1.

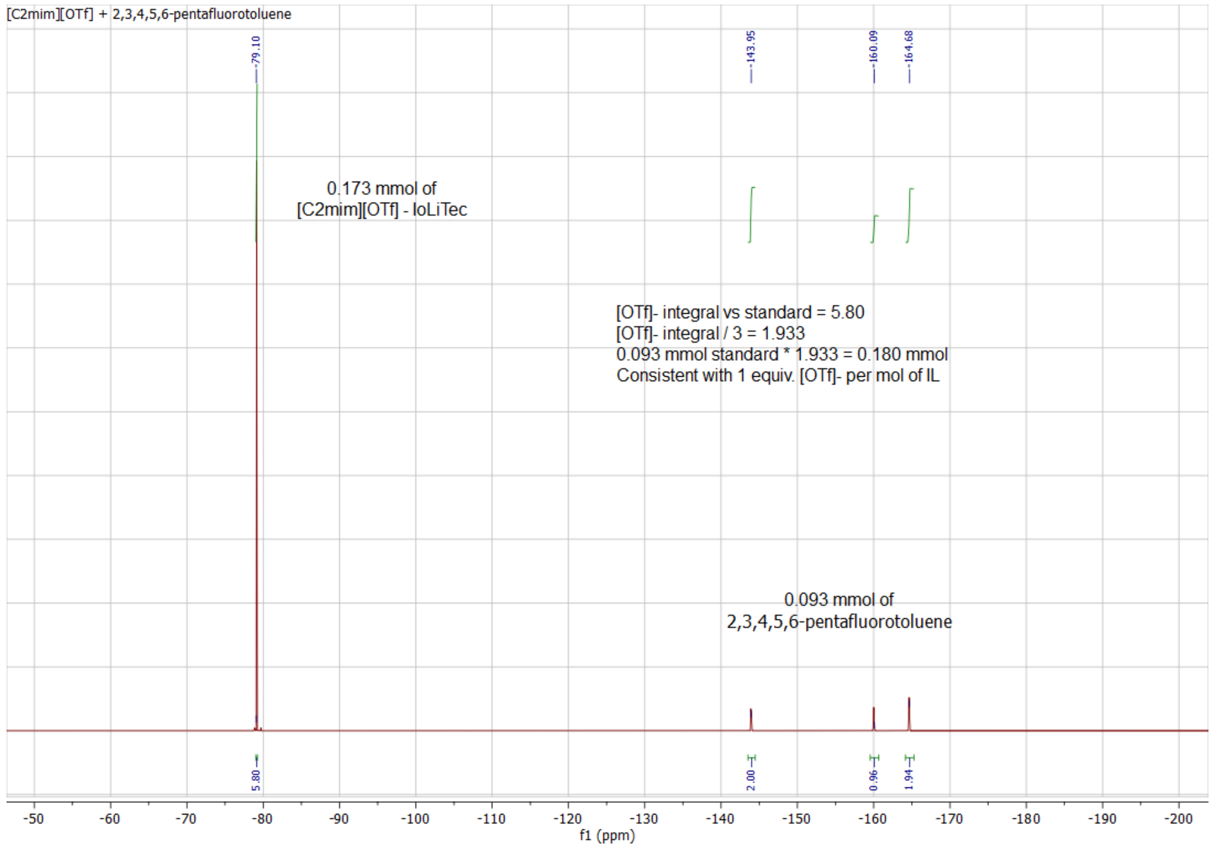


**Figure S1:** $^{19}$**F** NMR spectrum of [$C_2$mim][OTf] (from IoLiTec) including 2,3,4,5,6-pentafluorotoluene as an internal standard and integration confirming the presence of one $[OTf]^-$ anion per mole of IL, as expected.

**S2. RAS-LIF AlF yields from alternative samples of [$C_2$mim][OTf].**

There were no significant differences in the AlF appearance profiles from the two sources of [$C_2$mim][OTf], as shown in Fig. S2. The yields relative to [$C_2$mim][$Tf_2$N], using the same integration window as in the main text, were found to be 0.070 ± 0.006 and 0.062 ± 0.005 for the commercial (Iolitec) and self-synthesized samples, respectively.

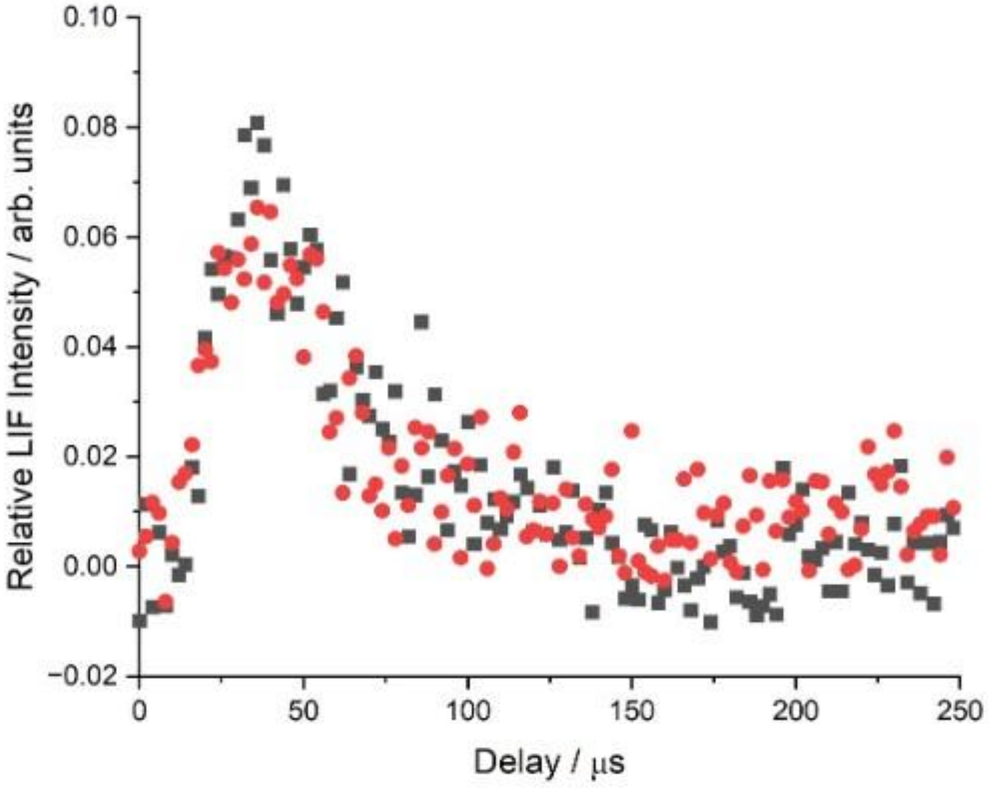


**Figure S2**: AlF appearance profiles from the two samples of [$C_2$mim][OTf]; commercial (black squares); self-synthesized (red circles). High fluence conditions, with ablation-pulse energy = 30 mJ.

## S3. Accessible surface areas from SASA and ball-drop methods.

Accessible surface areas were first determined by the standard SASA method, using a probe-particle of radius 0.18 nm to match the van der Waals radius of an Al atom. The ball-drop method was then applied to identify only those atoms that are directly accessible to a probe particle of the same radius approaching directly along the surface normal. The SASA-determined areas of these atoms was calculated. As can be seen in Table S1, the differences are very marginal. This confirms that, in this case, voids within the bulk of these liquids make a near-negligible contribution to the exposed surface areas.

**Table S1** Accessible areas as determined by the solvent-accessible surface area (SASA) method alone, compared with those obtained by filtering out any atoms determined not to be at the surface by the ball-drop method. Results are averaged over the final 10 ns of the equilibrated MD trajectory, as described in the main text. Errors represent the standard error of the mean between the 23 frames extracted from the trajectory.

| | **SASA** | | **Ball Drop and SASA** | |
|---|---|---|---|---|
| **Liquid** | **Area of all atoms / nm$^2$** | **Area of F-atoms / nm$^2$** | **Area of all atoms / nm$^2$** | **Area of F-atoms / nm$^2$** |
| [$C_2$mim][$Tf_2$N] | 131.9 ± 0.3 | 79.8 ± 0.3 | 131.7 ± 0.3 | 79.7 ± 0.3 |
| [$C_8$mim][$Tf_2$N] | 197.9 ± 0.5 | 32.9 ± 0.5 | 195.8 ± 0.5 | 32.5 ± 0.5 |
| [$C_2$mim][$BF_4$] | 97.6 ± 0.2 | 20.1 ± 0.2 | 97.4 ± 0.2 | 20.0 ± 0.2 |
| [$C_8$mim][$BF_4$] | 164.4 ± 0.6 | 4.7± 0.1 | 162.4 ± 0.5 | 4.5 ± 0.1 |
| [$C_2$mim][OTf] | 105.9 ± 0.3 | 55.6 ± 0.3 | 105.8 ± 0.3 | 55.5 ± 0.3 |
| [$C_8$mim][OTf] | 174.7 ± 0.5 | 14.3 ± 0.3 | 172.8 ± 0.4 | 14.1 ± 0.3 |
